# CBAM-YOLOv11 and Geometric Constraint-Enhanced PnP for High-Precision EV Charging Port Pose Estimation

**DOI:** 10.3390/s26175570

**Published:** 2026-09-02

**Authors:** Liangliang Wang, Mingming Lv, Qian Xu, Yuxi Cao

**Affiliations:** 1 School of Mechanical Engineering, Jiangsu University of Science and Technology, Zhenjiang 212003, China; 13775034183@139.com (L.W.); 17394791866@139.com (Q.X.); 2 School of Mechanical Engineering, Nanjing University of Science and Technology, Nanjing 210094, China; 3 School of Art and Design, Yangzhou University, Yangzhou 225009, China; bibingyan41@126.com

**Keywords:** EV charging, precise detection, pose estimation, YOLOv11, EPnP

## Abstract

The precise detection and pose estimation of electric vehicle (EV) charging ports in unstructured outdoor environments remain challenging due to small sizes, variable illumination, and stringent tolerance requirements for robotic plug-in operations. To address these issues, this paper presents a hybrid perception framework that integrates an attention-embedded detection network with geometrically constrained pose optimization. For robust detection, CBAM-YOLOv11 is proposed, which incorporates a sequential channel-spatial attention module into the backbone network to enhance feature representation of texture-less small targets while suppressing background clutter and glare. Then, a topological geometric constraint-based method is developed for accurate pose estimation. Specifically, the 2D-3D correspondences are purified before being fed into an Efficient Perspective-n-Point (EPnP) solver, while a nonlinear refinement with rigid distance priors is applied as regularization. Extensive experiments on the dataset and a physical robotic platform demonstrate that the proposed detector achieves 99.2% mAP@0.5 and a 24.6 percentage point improvement in mAP@0.5:0.95 over the baseline YOLOv11. The pose estimation module reduces positioning standard deviations along the X, Y, and Z axes to 4.72 mm, 5.65 mm, and 5.60 mm, respectively, surpassing conventional EPnP by about 60%. In 30 repeated robotic insertion trials, the system attains a 93.3% success rate with approximately 78 ms, fully satisfying real-time and precision requirements for autonomous EV charging.

## 1. Introduction

The global transition toward sustainable and low-carbon transportation has propelled the widespread adoption of electric vehicles (EVs) [1,2]. As EV penetration deepens, the demand for convenient and efficient charging solutions has become increasingly urgent. Despite being functionally adequate, traditional manual plug-in charging presents inherent limitations, particularly in adverse weather conditions, nighttime scenarios, shared parking facilities, and for users with reduced mobility [3,4]. More critically, the realization of a fully autonomous driving system inherently would benefit significantly from the seamless integration of autonomous charging capabilities. Robotic EV charging systems have thus emerged as a pivotal research frontier, offering the potential to enhance user experience, reduce operational costs, and achieve full automation in future mobility systems [5].

The fundamental technological bottlenecks in robotic EV charging lie in the accurate and robust perception of the charging port, which constitutes the essential prerequisites for subsequent robotic manipulation and precise plug-in operations. However, this task faces challenges for both the intrinsic properties of charging ports and the extreme variability of operational environments. The charging port typically belongs to a small object in the visual detection, which often occupies less than 100 × 100 pixels in standard images, and is overwhelmed by surrounding car-body textures and complex backgrounds [6]. At the same time, the outdoor lighting conditions are highly unpredictable and range from direct sunlight and glare to dim nighttime scenes. Furthermore, from a pose estimation perspective, the physical insertion of the charging plug demands exceptional precision. The typical alignment tolerances require positional errors of less than ±3 mm and orientational errors of less than ±3° to ensure further compliance with plug-in control without mechanical damage [3,7,8]. Achieving this precision within a real-time system presents a significant engineering challenge.

In response, substantial research efforts have been devoted to vision-based detection and pose estimation for EV charging ports. Early approaches relied on traditional image processing techniques, such as Hough transform and template matching [9,10]. Despite their computational efficiency, these methods exhibit poor robustness under varying illumination and partial occlusion. The advent of machine learning has revolutionized object detection, with the YOLO series emerging as a popular choice for real-time applications due to its superior trade-off between speed and accuracy [11]. Several studies have adapted YOLOv4 and YOLOv5 for charging port detection, which incorporate customized data augmentation strategies to enhance performance across diverse environments [12,13]. Specifically, YOLOv11 introduces C3K2 blocks, an enhanced spatial pyramid pooling fast module, and a C2PSA mechanism, which targets small-object detection and feature representation [14]. For instance, YOLOv11 has been successfully deployed for real-time axle type recognition from panoramic vehicle chassis images, which further demonstrates its effectiveness in complex vehicular perception tasks [15]. To focus more precisely on the charging port amidst complex backgrounds, attention mechanisms have been widely introduced into detection networks. The core of attention mechanisms lies in simulating human visual perception, enabling the network to automatically learn and focus on key features while suppressing irrelevant background information [16].

Recent studies have advanced attention-driven detection in challenging environments. For instance, Du et al. [17] proposed TSD-YOLO for small traffic signs, integrating a Space-to-Depth (SPD) module and Selective Kernel (SK) attention to preserve spatial details and adaptively adjust receptive fields for multi-scale objects. Similarly, Li et al. [18] developed Ihenet for low-light detection, employing an illumination-robust feature extractor (IRFE) and a hierarchical feature enhancement network (HFENet) to generate illumination-invariant features while preserving local details. Despite these advancements, their target scenarios and feature characteristics differ fundamentally from ours. TSD-YOLO focuses on structured road scenes, while Ihenet addresses general illumination variations. In contrast, our task involves detecting textureless, small targets in unstructured outdoor settings, where challenges extend beyond scale and illumination to include severe background clutter and strong specular reflections from metallic surfaces. Architecturally, while the aforementioned methods deploy attention modules across multiple feature levels or the entire network, our CBAM-YOLOv11 adopts a more targeted design. Guided by a theoretical analysis of receptive fields and semantic feature distribution, we strategically embed the CBAM exclusively at the P4 feature layer of the YOLOv11 backbone. This deliberate, localized placement achieves an optimal trade-off between computational efficiency and feature discriminability, distinguishing our approach from broad-attention methods.

For the pose estimation task, existing methods are classified into point cloud-based, monocular/stereo vision-based, and hybrid approaches [11,19]. Point cloud-based methods achieve high geometric accuracy through registration algorithms such as iterative closest point [20]. But due to leveraging 3D LiDAR or structured-light sensors, these methods suffer from high sensor costs and sensitivity to surface reflectivity. Monocular vision methods rely on perspective-n-point solvers with 2D-3D correspondences and offer cost-effective solutions [21,22]. However, they are inherently ill-posed and prone to ambiguity due to depth-scale uncertainties. Stereo vision methods address this limitation by actively triangulating depth from binocular disparity [23]. They enable more reliable metric reconstruction, but remain vulnerable to correspondence outliers and feature drift, particularly for texture-less objects like charging ports.

Despite these advances, there are some critical gaps in the visual detection and pose estimation of EV charging ports. First, attention mechanisms have been applied [24,25], but most implementations either deploy them indiscriminately across all feature levels or at overly deep layers where spatial resolution is already compromised, neither of which is optimal for small target detection. Second, conventional PnP-based methods treat 2D-3D correspondences independently without cross-verification and lack effective mechanisms to validate their mutual consistency [11]. This leaves them highly susceptible to mismatches introduced by glare, shadows, or edge artifacts, which inevitably contaminate the solution [20]. Third, the majority of prior works evaluate their methods solely on offline datasets or in purely simulated environments, without comprehensive system-level validation on physical robotic platforms that account for real-world constraints. Consequently, a practical solution remains conspicuously absent.

Thus, this paper presents a comprehensive and deployable framework for EV charging port perception and robotic guidance with an attention-enhanced detection network and a geometrically constrained pose optimization pipeline. Specifically, the convolutional block attention module (CBAM) is embedded into the P4 feature layer of the YOLOv11 backbone, named CBAM-YOLOv11. This deliberate placement balances computational efficiency and spatial information preservation, avoiding the high cost of shallow-layer attention while mitigating the excessive downsampling-induced loss of spatial detail in deeper layers. The sequential channel-spatial attention mechanism adaptively recalibrates feature responses and amplifies discriminative patterns such as arc contours and socket edges while actively suppressing background clutter and strong reflections. Thereby, robust detection under severe illumination variations is achieved.

For pose estimation, the geometrically constrained EPnP pipeline is developed, which exploits the inherent rigid topological relationships among charging port terminals as priors. The approach introduces a front-end geometric verification stage, which enforces distance, angle, and coplanarity consistency constraints. These constraints effectively purify 2D-3D correspondences before solving. Next, a nonlinear refinement is performed. It uses a weighted least-squares objective function, which embeds physical distance priors as regularization terms. Ultimately, this ensures both robustness and accuracy. Finally, comprehensive system-level validation is conducted on a physical experimental platform consisting of a 6-DoF collaborative robot, a binocular stereo camera, and a standard EV charging port assembly. The system integrates the proposed perception modules with real-time kinematic control, enabling closed-loop visual servoing. The experiments are performed under various conditions to evaluate detection precision, pose stability, real-time performance, and overall task success rate.

The remainder of this paper is organized as follows. Section 2 presents the proposed methodology in detail. Section 3 describes the experimental setup and presents comprehensive results and discussion. Finally, Section 4 concludes the paper and outlines future research directions.

## 2. Methodology

### 2.1. System Overview

**Figure 1:**
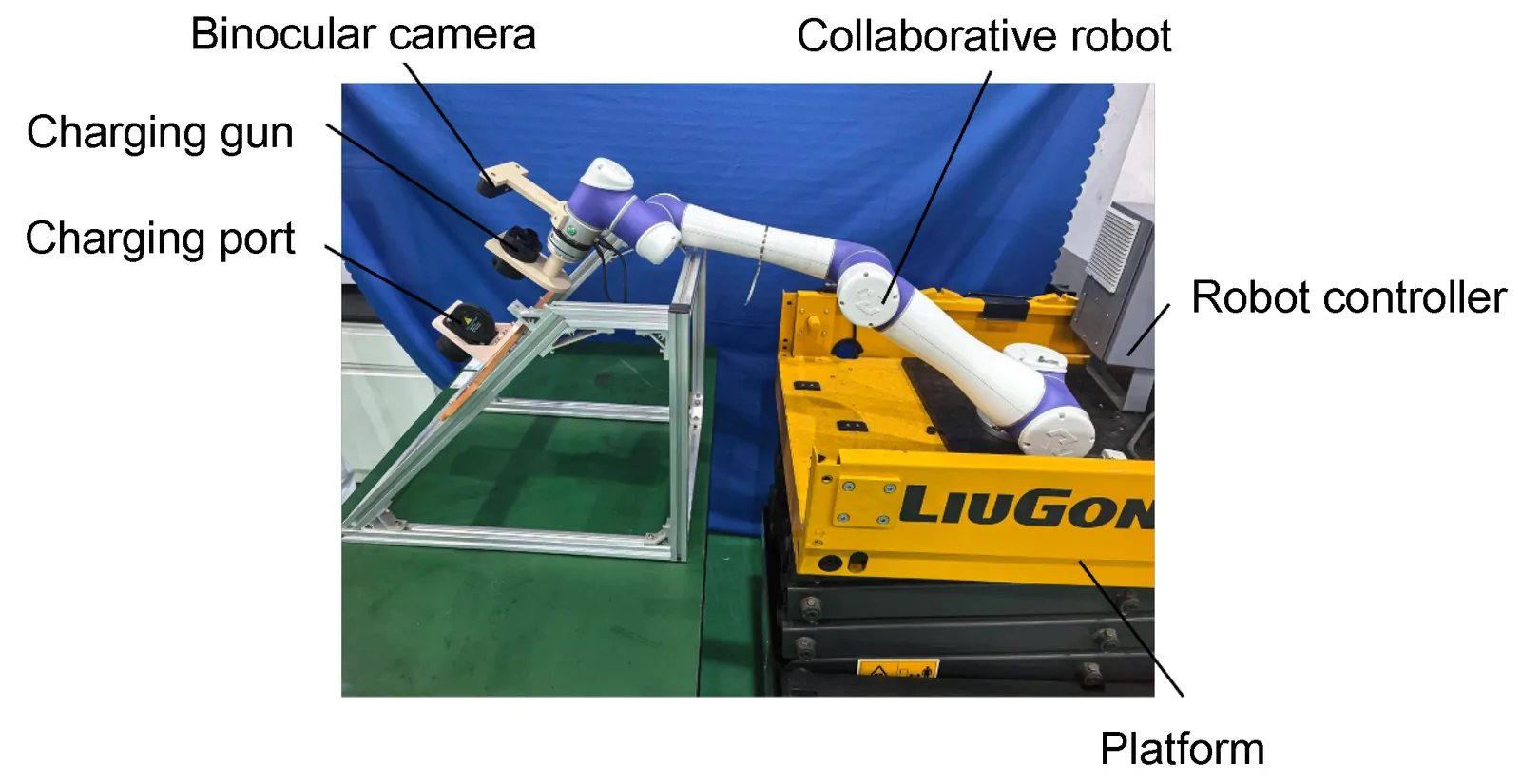
Hardware composition of the autonomous charging system. The black straight lines in the figure represent the leader lines.

The proposed robotic EV charging platform consists of a binocular stereo camera, a collaborative robot, and a charging gun, as illustrated in Figure 1. Specifically, the binocular stereo camera captures real-time images of the EV charging port to compute its 3D spatial position. The collaborative robot then receives this positional data, converts it into motion control commands, and achieves axial alignment between its end-effector and the EV charging port, which thereby lays the foundation for smooth insertion and subsequent docking.

In order to achieve precise guidance from the image pixel space to the robot workspace, the system has constructed a spatial transformation model that includes the pixel frame *O_uv_*, the camera frame *O_c_-X_c_Y_c_Z_c_*, the robot end-effector frame *O_e_-X_e_Y_e_Z_e_*, and the base frame *O_b_-X_b_Y_b_Z_b_*.

In the process, the stereo camera captures images of the EV charging port first, then the CBAM-YOLOv11 is employed to locate the region of the charging port. Combined with the triangulation principle, the physical depth *Z_c_* of the charging port in the *O_c_-X_c_Y_c_Z_c_* is directly calculated from the horizontal disparity between the left and right images with the stereo baseline distance. In practice, stereo disparity is computed using Semi-Global Block Matching (SGBM), followed by a Weighted Least Squares (WLS) filter to suppress edge noise. This enhances depth stability in weakly textured regions of the charging port. Based on the above depth computation, the depth map is pixel-wise aligned with the left RGB image via hardware-level Depth-to-Color (D2C) alignment, as natively supported by the Orbbec Gemini 2L camera, ensuring a one-to-one correspondence between each 2D pixel and its depth value. For any feature point (*u_i_*, *v_i_*), its 3D camera coordinate is then computed as *P_ci_* = *Z_ci_*·*K*^−1^·[*u_i_*, *v_i_*, 1]*^T^*, where *Z_ci_* = *f*·*b*/*d_i_* is obtained from the stereo disparity *d_i_* via triangulation, with *K* the intrinsic matrix, *f* the focal length, and *b* the stereo baseline. This enables accurate computation of the 3D Euclidean distance between any two points used in Equation (8). Subsequently, the 2D image coordinates (*u*, *v*) are back-projected into 3D camera coordinates using the intrinsic matrix and *Z_c_*. Next, the charging port is transformed from the frame *O_c_-X_c_Y_c_Z_c_* to the robot end-effector frame *O_e_-X_e_Y_e_Z_e_* using the hand-eye, which is obtained during offline calibration. Finally, by incorporating the real-time forward kinematics computed from joint encoders, this position is mapped to the frame *O_e_-X_e_Y_e_Z_e_*, which provides an absolute reference for the charging gun’s path planning and approach [26].

Synthesizing the aforementioned steps of stereo vision reconstruction and composite rigid transformations, the final mathematical model that converts the 2D pixel (*u*, *v*) into the absolute 3D base coordinate *P_b_* = [*X_b_*, *Y_b_*, *Z_b_*]^T^ is expressed as:

(1) XbYbZb1=Teb⋅Tce⋅(u−u0)⋅Zcfx(v−v0)⋅ZcfyZc1

where *u*_0_ and *v*_0_ represent the pixel coordinates of the principal point. Where *f_x_* and *f_y_* represent the camera’s pixel focal lengths along the horizontal and vertical axes. And Tce is a 4 × 4 rigid hand-eye that maps points from the camera frame to the robot end-effector frame, obtained via offline calibration and remaining constant throughout the process. Teb represents the 4 × 4 forward kinematics transformation matrix, which maps the end-effector frame to the base frame, and is computed in real-time by the teach pendant using current joint angles.

### 2.2. CBAM-YOLOv11 for Charging Port Detection

YOLOv11 employs an end-to-end framework. The backbone network leverages C3k2/C2f modules and SPPF for efficient multi-scale feature extraction and receptive field expansion. A neck with bidirectional pathways facilitates the deep fusion of cross-scale features, which are subsequently processed by a decoupled and anchor-free head. By integrating a TaskAligned strategy, the head ensures the direct generation of high-precision detection results [27,28].

However, the YOLOv11 exhibits significant limitations when detecting small objects such as charging ports. On one hand, due to the absence of specific constraints across spatial and channel dimensions, as the network depth increases, the sparse pixel information of the small charging port is easily overwhelmed by extensive vehicle body textures or complex background noise, preventing the model from effectively suppressing interference. On the other hand, the original network lacks sufficient capability to focus on critical local features, such as the subtle edges and contours of the internal socket, making it highly susceptible to missed detections and false positives when confronted with extreme lighting variations or significant angular tilts. Furthermore, during the multi-scale feature fusion stage within the network’s neck component, the absence of fine-grained attention weighting prevents the perfect alignment of deep-layer high-level semantic information with shallow-layer high-resolution positional information; this results in semantic ambiguity for low-resolution small objects during cross-scale concatenation, thereby significantly degrading the accuracy of the final bounding box localization [26,28].

**Figure 2:**
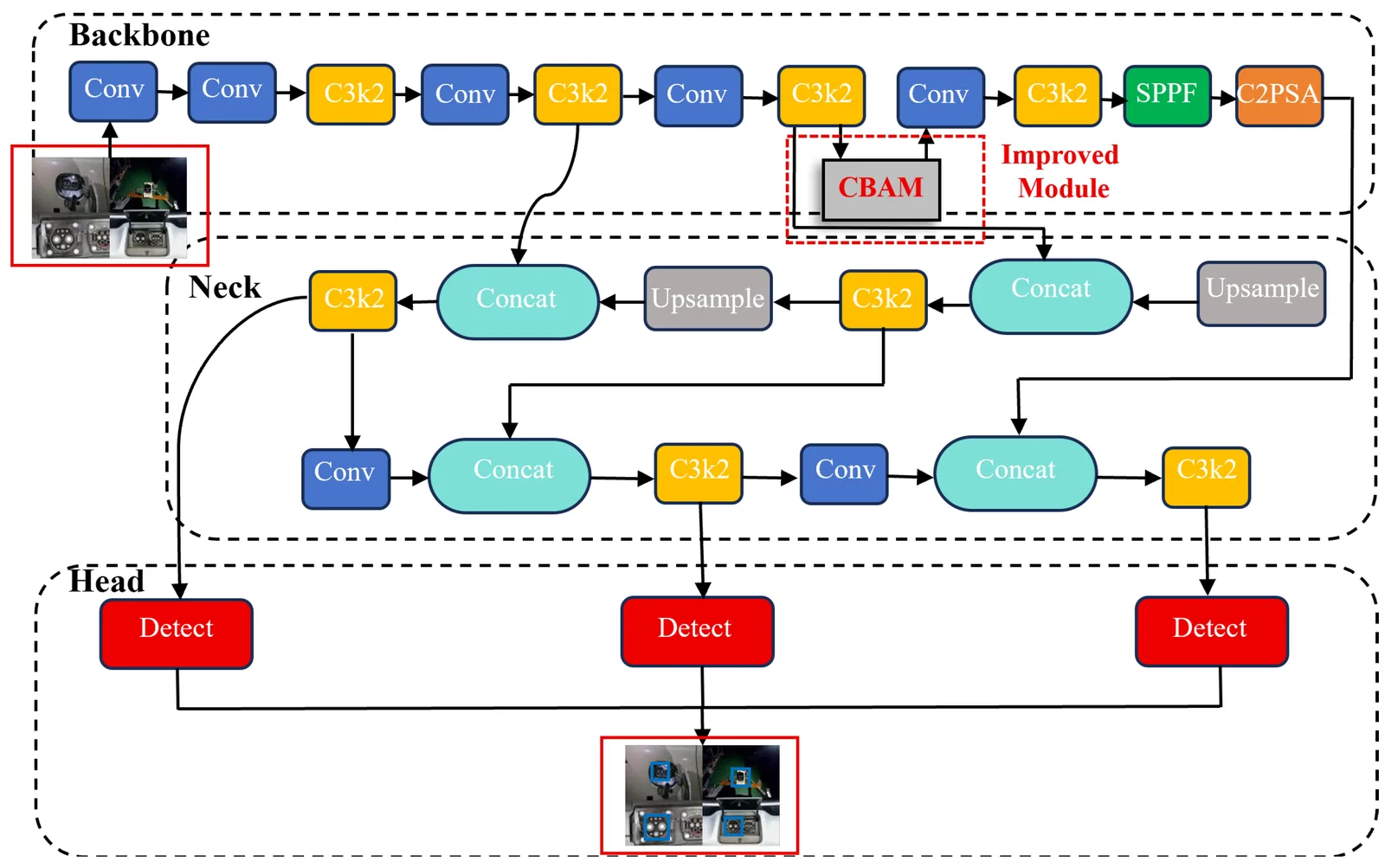
CBAM-YOLOv11 structure diagram. The red solid-line boxes represent the contents of our input and output, the red dashed-line boxes indicate the improvements we made to the YOLOv11 structure, and the blue solid-line boxes denote the recognized EV charging ports.

To address the challenges in electric vehicle charging port detection, such as strong light reflections on the car body and fine internal textures, this study embeds a CBAM attention mechanism into the P4 feature layer of the YOLOv11 backbone network, which serves as the transition from feature extraction to high-level semantics. The YOLOv11 structure after embedding CBAM is shown in Figure 2. The rationale for selecting the P4 feature layer is analyzed from the perspectives of receptive field and semantic feature distribution. The P3 feature map preserves abundant spatial details and fine textures because of its relatively high resolution. However, its receptive field is relatively limited, and the extracted features mainly represent low-level information such as edges and local textures. Under complex backgrounds or strong reflections, the channel attention mechanism at this stage may enhance not only charging-port features but also irrelevant background textures, thereby reducing feature discrimination. By contrast, the P5 feature map contains rich high-level semantic information and a sufficiently large receptive field to capture global contextual relationships. Nevertheless, the repeated downsampling process significantly decreases its spatial resolution, leading to the loss of structural details such as the circular boundary and internal holes of the charging port. This weakens the localization capability for small objects and limits the effectiveness of spatial attention. Compared with P3 and P5, the P4 feature map achieves a more appropriate balance between spatial resolution and semantic abstraction. Its receptive field is sufficiently large to model the global structure of the charging port while preserving adequate local geometric details for precise localization. Moreover, the feature channels at the P4 stage have evolved from low-level texture representations toward object-level semantic representations, enabling the channel attention mechanism to more accurately distinguish charging-port features from complex backgrounds. Subsequently, the spatial attention module further highlights the charging-port region while suppressing reflections, shadows, and background interference. Therefore, embedding the CBAM into the P4 layer provides an effective trade-off between semantic discrimination, spatial localization accuracy, and computational efficiency. This lightweight design avoids the computational overhead introduced by shallow-layer attention while preventing excessive spatial information loss caused by deep-layer downsampling. As a result, it significantly enhances feature representation capability with almost no increase in computational cost.

To mathematically demonstrate the coupling regulation process between this attention mechanism and the YOLOv11 backbone network, this paper reformulates the multi-level linkage dynamic calibration of dual attention into an end-to-end joint mapping composite equation:

(2) $$F^{\prime \prime }=\sigma \left(f^{7\times 7}\left(\left[AvgPool\left(Mc\left(F\right)⊗F\right);MaxPool\left(Mc\left(F\right)⊗F\right)\right]\right)\right)⊗\left(Mc\left(F\right)⊗F\right)$$

where *F* denotes the original feature map matrix input at the P4 stage. *F*″ is the final enhanced feature map. ⊗ represents element-wise multiplication between operators. The operator $M_{C}\left(F\right)=\sigma \left(W_{2}\left(\delta \left(W_{1}(F_{\mathrm{avg}})\right)\right)+W_{2}\left(\delta \left(W_{1}(F_{\max})\right)\right)\right)$ performs channel-wise selection of feature channels through element-wise per-channel multiplication. *F_avg_* and *F_max_* denote the one-dimensional channel feature vectors generated by global average pooling and global max pooling on the spatial dimensions of the feature map, respectively. *W*_1_ and *W*_2_ form the weight matrices of the shared fully connected layer in the multi-layer perceptron. *δ* denotes the Rectified Linear Unit activation function. *σ* represents the Sigmoid activation function.

**Figure 3:**
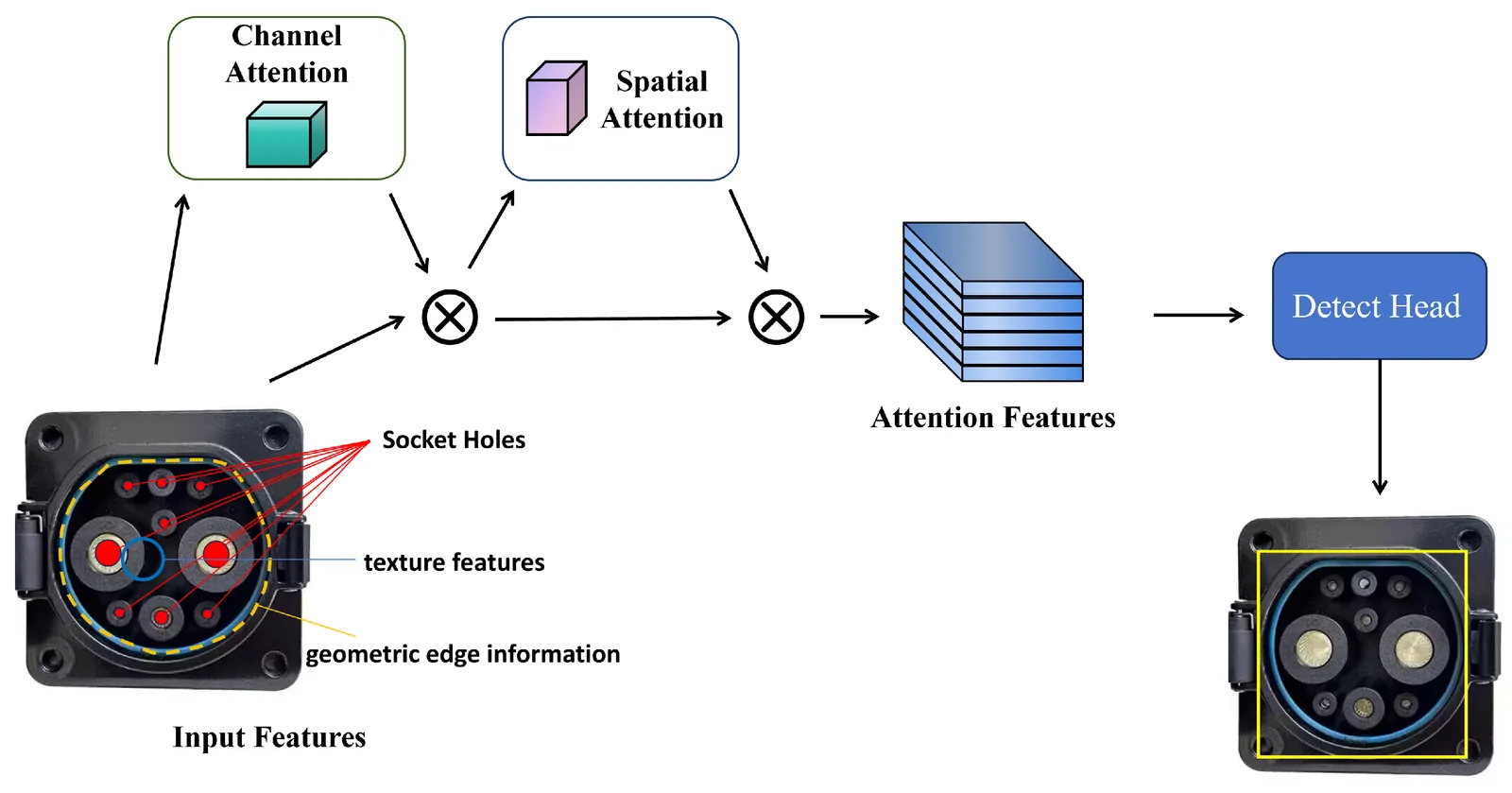
CBAM attention mechanism structure diagram. The yellow solid-line boxes denote the recognized EV charging ports. ⊗ represents element-wise multiplication of the attention weight map and the original feature map at each spatial position, thereby applying the attention weights to the features.

Subsequently, the intermediate state obtained through channel calibration is seamlessly fed into the second-stage spatial mapping operator, which consists of channel-wise concatenation and a large-kernel spatial convolution [29,30]. Here, *AvgPool*( ) and *MaxPool*( ) respectively denote the two-dimensional spatial plane features extracted after applying average pooling and max pooling along the channel dimension to the feature map. The spatial attention mechanism employs a *f* ^7×7^ large-kernel convolution with kernel size *k* = 7, using its broad receptive field to fully capture the global geometric contour information of the entire charging port as well as the relative spatial position relationship between the charging port and the surrounding car body environment. The two levels of attention operators work progressively; their structure is shown in Figure 3, and they finally output the enhanced feature map *F*″, achieving a substantial improvement in feature representation capability with almost no increase in computational cost. This significantly alleviates the problems of missed detections and false alarms for the target under complex vehicle-environment interference, laying a solid feature foundation for the subsequent high-precision bounding box regression of the anchor-free decoupled detection head and binocular 6D pose estimation.

### 2.3. 3D Localization and Pose Estimation

To enable the collaborative robot to autonomously and precisely insert and extract the EV charging connector, it is essential to obtain its real-time, high-accuracy 6-DoF pose relative to the camera [31].

To address this, we propose a 3D localization and pose estimation framework based on the synergy of topological geometric constraints and robust EPnP. Unlike traditional methods that rely solely on the standard EPnP closed-form solution [32], this method incorporates the charging port’s rigid topological geometry as a core prior. This enables a robust perception closed-loop comprising three stages: front-end geometric matching and purification, back-end efficient closed-form computation, and nonlinear iterative refinement.

Within this framework, the classic EPnP serves as the underlying solver for the system of linear equations. Fundamentally, it represents the *n* 3D feature points on the charging port as a weighted combination of four virtual control points. This formulation reduces the complex absolute pose estimation problem to solving for the *n* 3D coordinates of these control points in the camera frame.

**Figure 4:**
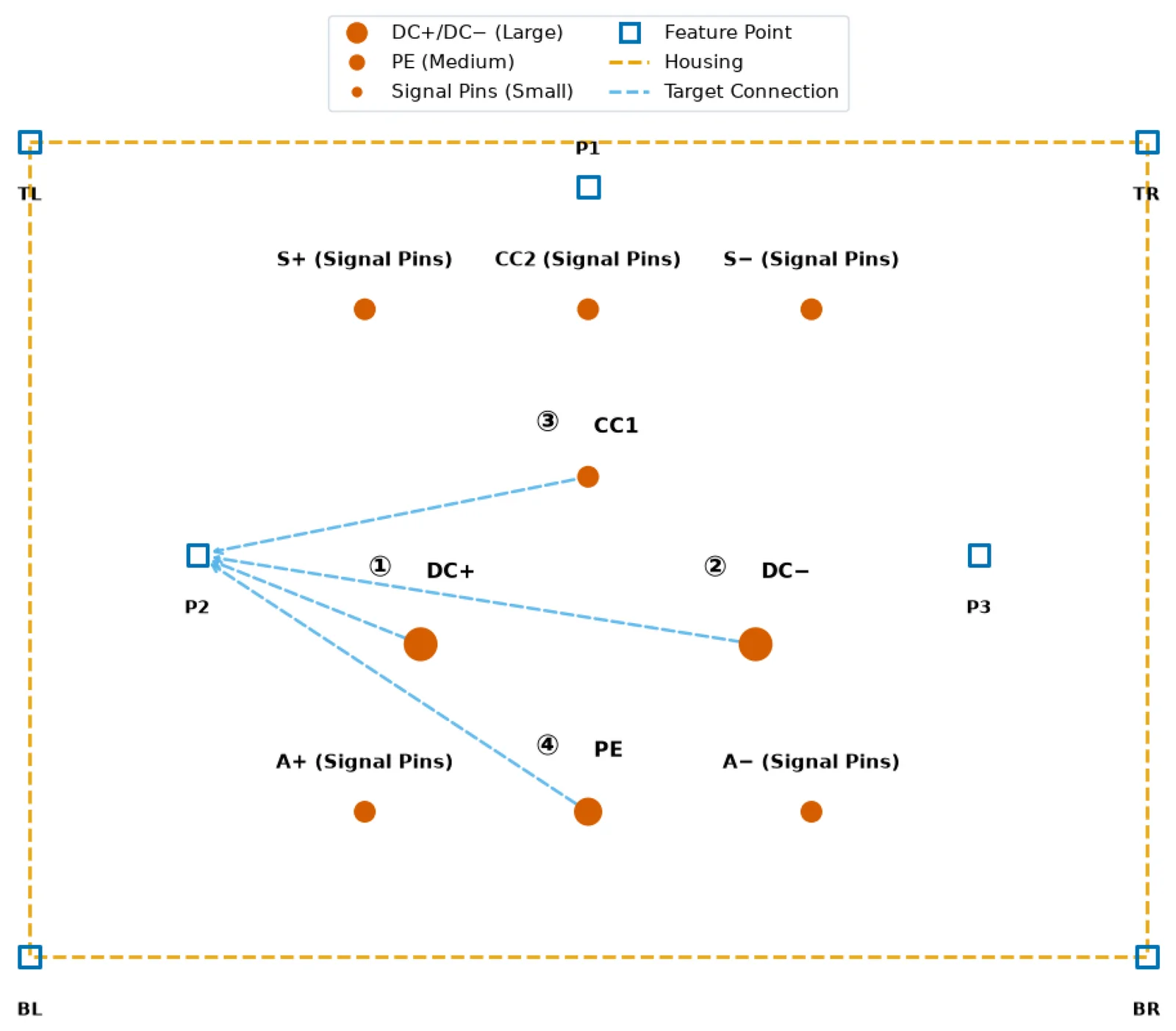
Schematic diagram of virtual control point construction and its topological mapping relationship with physical feature points. The numbered labels in the figure indicate the four core keypoints, which are used as virtual control points.

Let $P_{i}^{w}$ = [$X_{i}^{w}$, $Y_{i}^{w}$, $Z_{i}^{w}$]*^T^* (for *i* = 1, …, *n*) denote the coordinates of the known 3D feature points on the charging port in the world frame. Similarly, let $C_{j}^{w}$ = [$X_{j}^{w}$, $Y_{j}^{w}$, $Z_{j}^{w}$]^T^ (for *j* = 1, 2, 3, 4) represent the four selected virtual control points in the same coordinate system. The geometric linear combination relating them is defined as [33]:

(3) $$P_{i}^{w}=\sum_{j=1}^{4}\alpha_{ij}C_{j}^{w}s.t.\sum_{j=1}^{4}\alpha_{ij}=1$$

where $\alpha_{ij}$ represents the homogeneous barycentric coordinate of the *i*-th feature point with respect to the *j*-th control point, and *n* represents the total number of 3D feature points involved in the computation. Since this coefficient relies solely on the intrinsic geometry of the charging port, it can be computed during an offline preprocessing stage. Furthermore, as visualized in Figure 4, due to the linearity of rigid body transformations, this virtual control point formulation reduces the 6D pose estimation of arbitrary n feature points into determining the 3D spatial coordinates of the 4 virtual control points, significantly improving computational efficiency and resistance to single-point extraction outliers. This linear combination property remains invariant in the camera coordinate system:

(4) $$P_{i}^{c}=\sum_{j=1}^{4}\alpha ijC_{j}^{c}=\sum_{j=1}^{4}\alpha ij\left[\begin{array}{c} X_{j}^{c}\\ Y_{j}^{c}\\ Z_{j}^{c} \end{array}\right]$$

where $P_{i}^{c}$ denotes the 3D coordinates of the feature point in the camera frame, while $C_{j}^{c}$ represents the unknown 3D coordinates of the virtual control points to be estimated. And $\alpha_{ij}$ is the same as the homogeneous barycentric coordinates in Equation (3).

Based on the pinhole camera model and the linear representation of control points, the pixel projection equation for each feature point is derived as follows:

(5) $$Z_{i}^{c}\left[\begin{array}{c} u_{i}\\ v_{i}\\ 1 \end{array}\right]=KP_{i}^{c}=K\sum_{j=1}^{4}\alpha_{ij}C_{j}^{c}$$

where Pi = [*u_i_*, *v_i_*]^T^ denotes the 2D pixel coordinates of the feature point, and *K* represents the known camera intrinsic matrix. The term $Z_{i}^{c}$ corresponds to the depth of the feature point along the optical axis in the camera frame. By aggregating all *n* feature points and eliminating the unknown depth terms $Z_{i}^{c}$, a linear homogeneous system of the form *Mx* = 0 is constructed. Here, the unknown vector *x* = [($C_{1}^{c}$)^T^, ($C_{2}^{c}$)^T^, ($C_{3}^{c}$)^T^, ($C_{4}^{c}$)^T^]^T^ comprises the 12 unknown 3D coordinates of four control points in the camera coordinate system.

This paper presents a geometrically constrained feature matching method integrated into the pose estimation front-end, serving as a critical mechanism to ensure the robustness of the solver. The core logic of this method is to utilize the inherent rigid topological relationships of charging port terminals in 3D physical space as strong prior constraints, without disrupting the efficient linear closed-form solution structure of the traditional EPnP. Specifically, strict geometric constraint enforcement and purification are applied to the set of 2D candidate feature points prior to constructing the *M* matrix.

The physical terminal centers of a standard charging port exhibit invariant geometric topological relationships within the rigid three-dimensional space. Consequently, the Euclidean distance prior between any two feature points, denoted as *X_i_* and *X_j_*, in the world coordinate system is defined as:

(6) $${D_{ij}\;=\;\left\|X_{i}-X_{j}\right\|}_{2}$$

A fully connected geometric topology graph is constructed from the candidate feature points extracted by the front-end detection network. For any pair of corresponding edges, their lengths and angles must satisfy the consistency constraints imposed by rigid transformations in 3D space. Distance consistency constraint: Utilizing the feature point depths preliminarily estimated by the binocular vision system or the relative distances in the normalized camera coordinate system, we verify that the deviation between the predicted spatial distance of the current 2D point pair and the physical prior satisfies the inequality $\left\|D_{ij}^{\prime }-D_{ij}\right\|<\varepsilon d$, where *ε_d_* denotes the distance error threshold. Angle and collinearity constraints exploit the multi-point coplanarity of the charging port terminals to reject outliers that deviate significantly from the planar projection geometry.

Only those 2D-3D point pairs that simultaneously satisfy the aforementioned topological consistency of spatial rigid geometric constraints are classified as correct matches. Through this mechanism, the system effectively prunes and filters out all mismatched points caused by reflections or shadows at the front end.

Following the purification process using the aforementioned geometric-constrained feature matching method, all inputs to the core solver consist of high-purity correct point pairs. Based on the homogeneous linear projection mapping between the world and camera coordinate systems for virtual control points, a homogeneous linear equation system *Mx* = 0 is constructed, where *M* denotes the coefficient matrix, and *x* represents the unknown vector of control point coordinates. The closed-form solution to this system can be accurately obtained by computing the null-space eigenvectors of the purified coefficient matrix *M*:

(7) $$x=\sum_{i=1}^{n}\beta_{i}v_{i}$$

where *x* = [($C_{1}^{c}$)^T^, ($C_{2}^{c}$)^T^, ($C_{3}^{c}$)^T^, ($C_{4}^{c}$)^T^]^T^ is the 12-dimensional unknown vector composed of the coordinates of the four virtual control points expressed in the camera coordinate system, and *v_i_* denotes the eigenvector within the null space of the coefficient matrix *M*, while *β_i_* represents the unknown combination coefficients to be determined. By enforcing the rigid geometric constraint that inter-point distances remain invariant, *β_i_* is resolved, thereby recovering the absolute coordinates of the four control points in the camera coordinate system. Subsequently, the initial rotation matrix *R*_0_ and translation vector *T*_0_ of the charging port relative to the camera are efficiently derived via Singular Value Decomposition (SVD).

To achieve the high-precision positioning required for robotic arm-guided insertion and extraction, this work deviates from the traditional reprojection error minimization. Instead, it incorporates the aforementioned rigid geometric constraint as a regularization term within the objective function. Consequently, a nonlinear iterative refinement model based on weighted least squares is formulated as follows:

(8) $$\underset{R,T}{\min}\left(\sum_{i}w_{i}{\left\|u_{i}-P\left(R\cdot p_{i}^{w}+T\right)\right\|}_{2}^{2}+\lambda {\sum_{i,j}\left({\left\|P^{-1}\left(u_{i}\right)-P^{-1}\left(u_{j}\right)\right\|}_{2}-D_{ij}\right)}^{2}\right)$$

where the decision variables *R* and *T* denote the optimized 3D rotation matrix and translation vector, respectively. Within the standard reprojection error term, *w_i_* serves as a weight coefficient dynamically configured according to front-end feature matching confidence to reflect the extraction accuracy of specific keypoints. Here, *u_i_* represents the homogeneous coordinates of the observed keypoint in the image plane, *P* is the nonlinear camera projection function, and $p_{i}^{w}$ denotes the prior 3D coordinate vector in the world frame; the term ${\left\|\cdot \right\|}_{2}^{2}$ indicates the squared Euclidean norm of the residual. Regarding the geometric constraint term, *λ* acts as a regularization parameter that balances the reprojection error against physical constraints. The function *P*^−1^ represents the back-projection mapping, which transforms the 2D observation *u_i_* into the 3D camera space using the inverse intrinsic matrix *K*^−1^ and binocular depth information. Finally, ${\left\|P^{-1}\left(u_{i}\right)-P^{-1}\left(u_{j}\right)\right\|}_{2}$ denotes the computed 3D Euclidean distance between keypoints *i* and *j* under the current pose, while *D_ij_* represents the inherent ground-truth distance prior of the standard charging port features. The objective function in Equation (8) is inherently non-convex due to the nonlinear camera projection model within the reprojection error term. Consequently, a global minimum cannot be guaranteed, rendering the optimization vulnerable to local minima. To mitigate this risk, our framework employs a two-pronged strategy. First, the closed-form EPnP solution (R_0_, T_0_) serves as a robust initialization, positioning the solver in close proximity to the true pose. Second, our front-end geometric verification enforcing strict distance and coplanarity constraints ensures that only high-quality inlier correspondences are fed into the optimization process.

**Figure 5:**
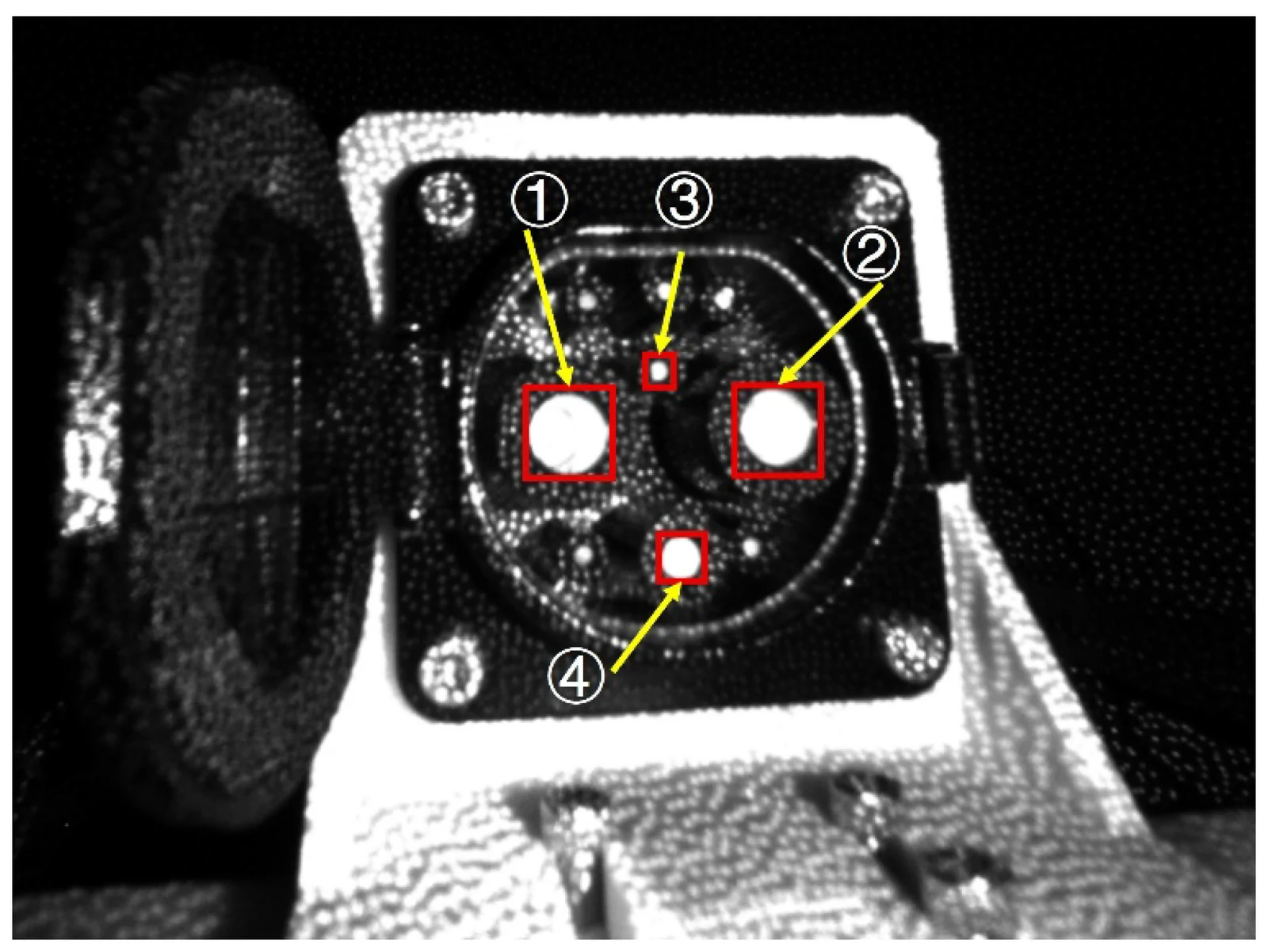
The four core physical keypoints (①–④) serve as both the virtual control points in the EPnP formulation (Equations (3)–(5)) and the 2D-3D correspondences for pose estimation. The remaining unannotated holes are used for post-optimization verification. The red solid-line boxes indicate the four virtual control points.

As illustrated in Figure 5, the annotated keypoints ①, ②, ③, and ④ constitute the core set for geometric computation and constraint. According to the national standards (GB/T) regulating EV charging interfaces, the geometric centers of these four core apertures form a rigid structure with strictly invariant pairwise relative distances in 3D space. During pose estimation, the algorithm aligns the 2D image projections of these apertures with their corresponding points in the known 3D model. Through nonlinear optimization, the rotation matrix and translation vector are iteratively refined to minimize the deviation between the spatial distances derived from the current pose and the ground-truth physical dimensions.

In our framework, the four core apertures (①–④) serve dual roles: they are the physical keypoints providing 2D-3D correspondences, and simultaneously the virtual control points in the EPnP formulation. The number of keypoints required is K = 4, which is the minimum needed for the EPnP solver. The geometric constraints in Equation (6) and the regularization term in Equation (8) are applied exclusively to these four core apertures, with fixed distance priors derived from the GB/T standard. The remaining unannotated verification holes are used solely for post-optimization consistency checking to validate the estimated pose and reduce the risk of local optima.

To further validate the pose estimation accuracy and mitigate the risk of convergence to local optima, this method utilizes the remaining unannotated feature holes to establish a consistency check set. Upon obtaining the initial pose, the algorithm reprojects the 3D feature holes from the check set onto the 2D image plane, computing the reprojection error relative to the detected pixel centers. This closed-loop mechanism, employing four holes for high-precision solving and the remainder for cross-validation, not only suppresses anomalies induced by illumination variations and edge distortion but also significantly enhances system safety and robustness during automatic charging docking.

The linear closed-form solution (*R*_0_, *T*_0_) serves as the initial guess for the subsequent nonlinear refinement of the composite objective function in Equation (8), which is implemented via the Levenberg–Marquardt algorithm. During the iterative optimization, the rotation matrix *R* is parameterized as a three-dimensional Rodrigues vector r = [*r*_1_, *r*_2_, *r*_3_]*^T^*, which is converted to a 3 × 3 rotation matrix at each iteration through the Rodrigues transformation. This parameterization intrinsically guarantees the orthogonality constraint R^T^R = I and det(R) = 1, without directly optimizing the nine matrix elements. Since the preceding geometrically constrained matching guarantees that all points involved in the optimization are inliers, the reprojection error surface is exceptionally smooth and free from gross errors. Consequently, the nonlinear optimization converges rapidly within merely three to five iterations.

## 3. Experimental Results

### 3.1. Experimental Setup

**Figure 6:**
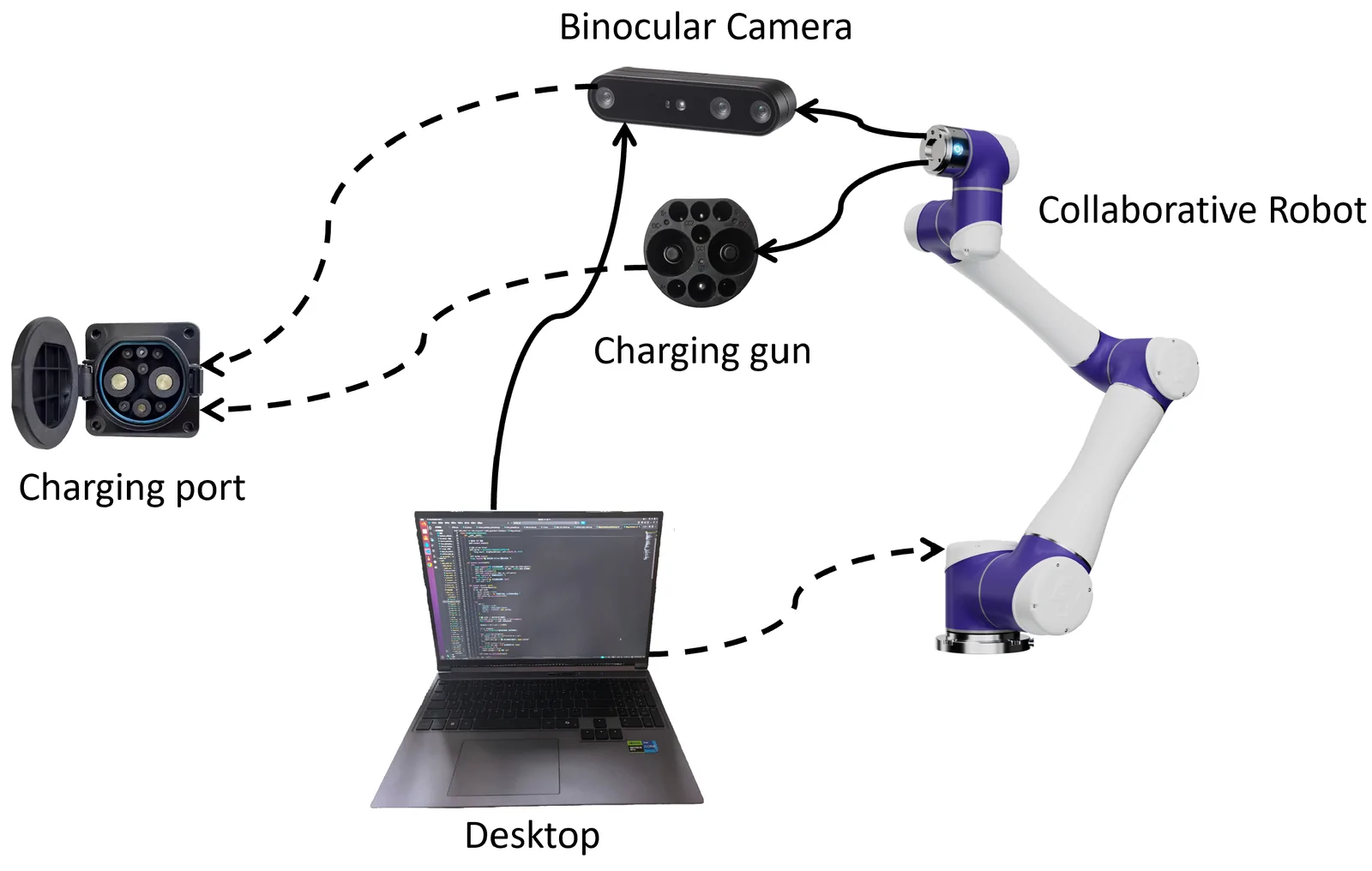
Schematic diagram of the autonomous EV charging experimental platform. It consists of a 6-DoF collaborative robot, a stereo depth camera, the charging gun and port, and the desktop. Solid lines represent required physical connections between devices, while dashed lines indicate no physical connection is needed between devices.

To verify the effectiveness and robustness of the proposed method in real-world scenarios, a hardware experimental platform is established, which primarily consists of an Elite EA66 6-DoF collaborative robot (Elite Robots, Suzhou, Jiangsu, China), an Orbbec Gemini 2L binocular stereo camera (Orbbec, Shenzhen, Guangdong, China), a standard EV charging port assembly (GB/T 20234.3 [34]-compliant DC charging port), and a high-performance laptop, as shown in Figure 6. The robot is utilized for its high-precision motion control capabilities (repeatability: ±0.05 mm), suitable for simulating complex plug-in operations, while the binocular stereo camera is mounted on the robot end-effector in an eye-in-hand configuration, responsible for capturing environmental images and acquiring depth information to support visual servoing. The Orbbec Gemini 2L camera features active stereo IR technology with a 100 mm baseline, a depth range of 0.2–10 m, and RMSE < 2% at 4 m. The depth resolution is 1280 × 800 @ 30 fps (FoV: 91°H × 66°V), and the RGB resolution is 1280 × 800 @ 30 fps (FoV: 94°H × 68°V).

The software system is built upon the Linux-Ubuntu 20.04 operating system (Canonical Ltd., London, United Kingdom). The experimental desktop is equipped with an Intel Core i7-13700 CPU (Intel Corp., Santa Clara, CA, USA) and an NVIDIA GeForce RTX 5060 GPU (16 GB VRAM) (NVIDIA Corp., Santa Clara, CA, USA), utilizing the CUDA 13.0 architecture (NVIDIA Corp., Santa Clara, CA, USA) to accelerate the inference and training of deep neural networks. The deep learning environment is implemented using the PyTorch 2.9.1 framework (Meta, Menlo Park, CA, USA). During the model training phase, hyperparameters were optimized to ensure detection accuracy and convergence speed. The specific training configurations are detailed in Table 1.

### 3.2. Detection Performance Evaluation

#### 3.2.1. Dataset Construction

A hybrid dataset is constructed by integrating publicly available images with custom-captured EV charging port photographs [35]. The proprietary images are acquired across diverse real-world scenarios, including underground parking lots and open-air stations, utilizing an Orbbec Gemini 2L binocular stereo camera and a high-resolution smartphone to encompass varied vehicle models, viewing angles, and illumination conditions. The dataset comprises over 2000 annotated images.

Ground-truth bounding boxes are meticulously annotated via the Roboflow platform. Prior to model training, all images underwent standardized preprocessing, including automatic orientation correction and resizing to a uniform resolution of 640 × 640 pixels. To bolster generalization and mitigate overfitting, a comprehensive data augmentation pipeline is implemented. This included random shearing (±10° horizontally and vertically) to simulate perspective shifts, photometric adjustments (hue, brightness, and contrast) to replicate extreme lighting, and the injection of Gaussian blur and noise to mimic lens contamination. Finally, the dataset was strictly partitioned into training, validation, and test sets at a ratio of 7:2:1.

#### 3.2.2. Ablation Study

To elucidate the contribution of individual components and the underlying mechanism of CBAM in charging port detection, ablation studies are conducted using YOLOv11 as the baseline, as summarized in Table 2. The results demonstrate that each attention module independently enhances detection performance. Specifically, integrating SAM increases mAP@0.5:0.95 to 66.2%, confirming its efficacy in capturing spatial contour and edge features. Incorporating CAM further elevates mAP@0.5:0.95 to 74.8%; this indicates that channel attention establishes global dependencies to amplify intrinsic texture features of the EV charging port while effectively suppressing environmental interference such as glare and shadows.

In ablation experiments on the insertion position, when CBAM is placed at the neck or the detection head, the performance improvement is limited due to the attenuation of spatial information caused by downsampling of deep feature maps. In contrast, embedding CBAM into the backbone network achieves the global optimum, with mAP@0.5 reaching 99.2%, and the strict localization metrics mAP@0.75 and mAP@0.5:0.95 significantly improving by 26.2 percentage points and 24.6 percentage points over the baseline, respectively. This demonstrates that introducing dual attention calibration at the transitional layers of the backbone can effectively prevent the loss of deep pixel information, thereby laying a solid feature foundation for subsequent high-precision pose estimation. These findings confirm that dual attention calibration at early feature extraction stages mitigates pixel-level information loss in deep networks, thereby establishing a robust feature foundation for high-precision pose estimation under complex sensing conditions.

#### 3.2.3. Comparison Study

**Figure 7:**
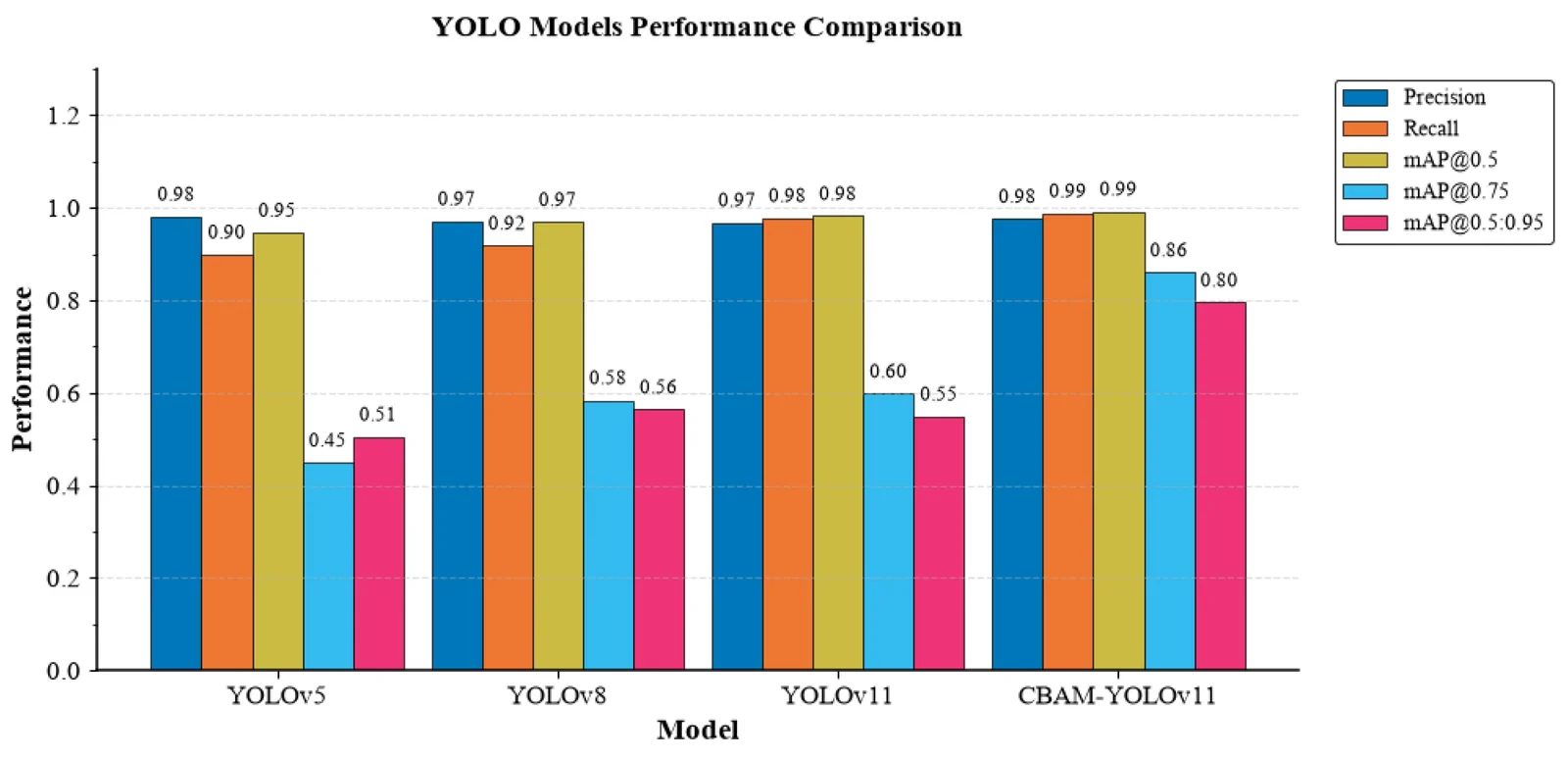
Comparative analysis of detection performance between the proposed CBAM-YOLOv11 and other state-of-the-art models. The evaluation metrics include Precision, Recall, and mAP at different IoU thresholds.

To validate the effectiveness of the proposed CBAM-YOLOv11, comparative experiments were conducted against YOLOv5, YOLOv8, and the baseline YOLOv11. As illustrated in Figure 7, the proposed model exhibits superior performance across multiple key metrics. Notably, CBAM-YOLOv11 achieves a detection rate of 97.6% and mAP@0.5 of 99.2%, representing a marginal improvement over the baseline while maintaining high precision and recall. More significantly, it demonstrates substantial gains in stricter localization metrics, with mAP@0.75 and mAP@0.5:0.95 increasing by 26.2 percentage points and 24.6 percentage points, respectively, compared to the original YOLOv11. These results confirm that the integration of CBAM effectively enhances localization robustness against environmental disturbances.

### 3.3. Pose Estimation Accuracy

**Figure 8:**
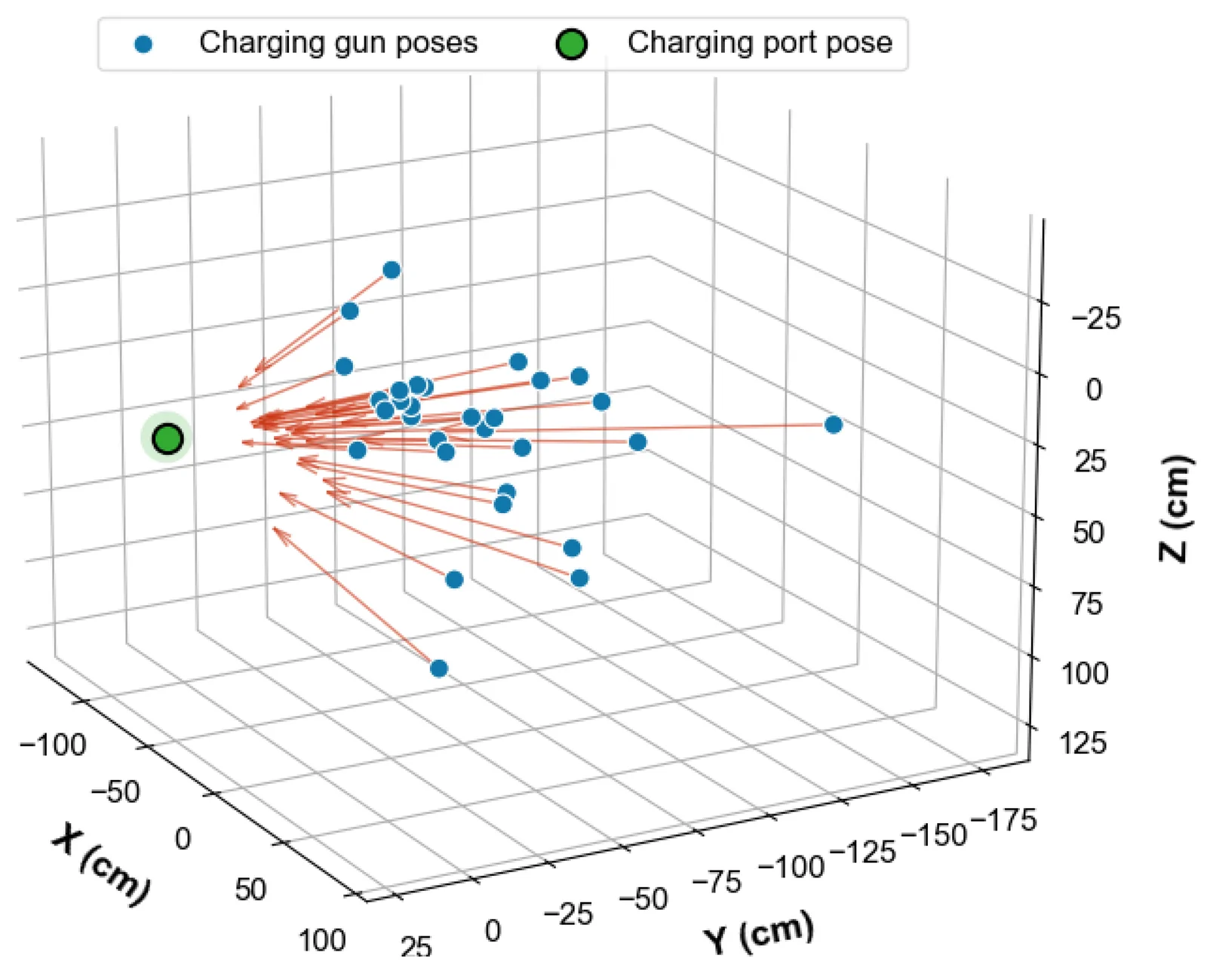
Spatial distribution of the charging gun poses (blue balls) relative to the charging port (green ball). The red arrows indicate the orientation vectors pointing towards the target.

To evaluate the accuracy and stability of the proposed geometrically constrained EPnP method, repeated pose estimation experiments are conducted on a charging port across 30 distinct charging gun configurations. In each trial, the 6-DoF pose is computed using the proposed algorithm and subsequently transformed into the robotic coordinate system via hand-eye calibration and forward kinematics. The ground-truth pose of the charging port was obtained by manual measurement using high-precision calipers and protractors, ensuring independence from the vision-based estimation (see Section 3.4). Figure 8 illustrates the spatial distribution of the charging gun across these 30 positions, alongside the charging port location within the world frame.

**Figure 9:**
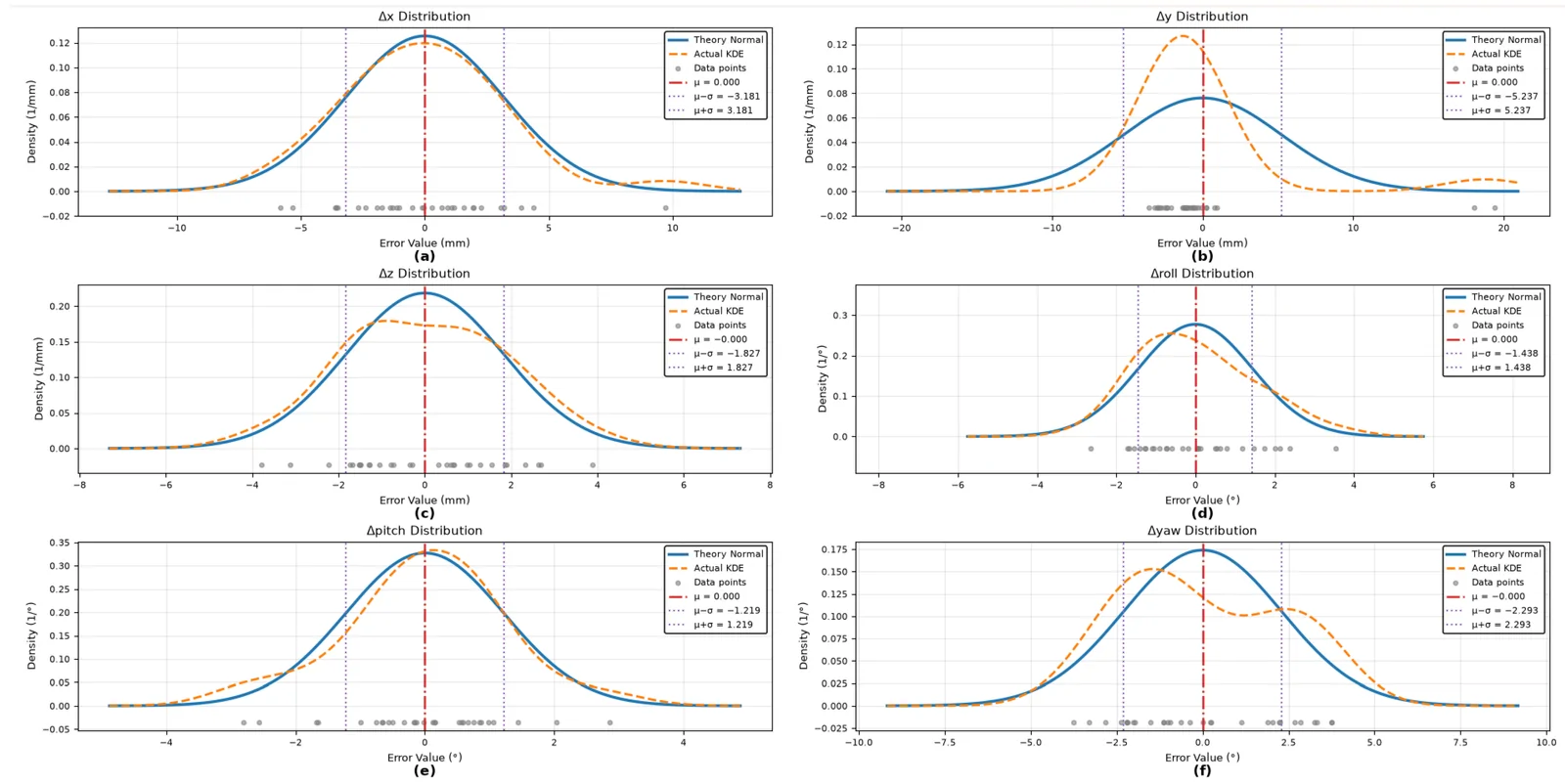
Statistical analysis of the 6-DoF pose estimation errors across 30 trials. The plots display the probability density functions for translational (**a**–**c**) and rotational (**d**–**f**) errors, demonstrating a close fit to zero-mean Gaussian distributions.

Figure 9 illustrates the 6-DoF error distributions across the 30 trials. Both translational and rotational errors closely follow zero-centered normal distributions, as evidenced by the high consistency between the actual data histograms and the theoretical Gaussian curves. This confirms the absence of significant systematic bias and demonstrates consistent stability under varying viewing angles. Furthermore, the concentrated error spread validates that the topological geometric constraints effectively enhance pose estimation robustness.

Table 3 presents the 95% confidence intervals (CIs) for the 6-DoF pose estimation errors of the proposed method, computed over 30 independent trials. All intervals contain zero, statistically confirming the absence of significant systematic bias-a finding consistent with the zero-mean Gaussian distributions observed in Figure 9. The CI widths vary considerably across the six degrees of freedom: the narrowest interval is observed for Δroll (0.596°), benefiting from the strong geometric constraints imposed by the horizontally distributed feature points of the charging port, whereas Δpitch exhibits the widest interval (1.496°), reflecting the inherent limitation that pitch estimation relies primarily on depth measurements and is therefore susceptible to disparity quantization errors. For the translational components, the CIs are Δx: ±1.69 mm, Δy: ±2.02 mm, and Δz: ±2.00 mm-all falling well within the ±3 mm tolerance threshold, indicating that even at the 95% confidence level, the mean estimation errors strictly satisfy the precision requirements for robotic plug-in tasks. Furthermore, the difference in interval widths between Δx (3.38 mm) and Δy (4.04 mm) reflects the anisotropy of the charging port’s feature point layout, where the larger horizontal span provides stronger geometric constraints.

For quantitative benchmarking, the traditional EPnP algorithm and OPnP—a globally optimal, non-iterative PnP solver known for its improved robustness to noise and outliers compared to the closed-form EPnP solution—were both evaluated as baselines under identical experimental conditions, utilizing the same 2D feature points, camera intrinsics, and 30 robot arm configurations. The primary distinction of the proposed method lay in the incorporation of geometric constraints. Table 4 summarizes the 6-DoF standard deviations for all three methods. Compared to the baseline EPnP, which exhibited translational errors of 16.6 mm (X), 15.5 mm (Y), and 8.1 mm (Z), OPnP achieved moderately lower errors of 11.2 mm, 10.8 mm, and 6.9 mm, respectively, confirming that a more robust solver alone yields partial improvement. Nevertheless, the proposed method further reduced these values to 4.72 mm, 5.65 mm, and 5.60 mm, respectively, alongside concurrent improvements in rotational stability, outperforming both baselines. These results confirm that the geometric constraint mechanism rather than the PnP solver itself is the dominant factor in mitigating feature matching disturbances caused by reflections, occlusions, and complex backgrounds, thereby significantly enhancing pose estimation accuracy and robustness.

It is noteworthy that the baseline EPnP exhibits the smallest error along the Z-axis, which appears contrary to the typical behavior of stereo-based pose estimation where depth uncertainty usually dominates. This is not because the baseline achieves better Z-axis accuracy, but rather an artifact caused by outlier contamination: without front-end verification, mismatched correspondences force the least-squares solver to redistribute large reprojection residuals over the X and Y translation components, making the Z-axis error appear comparatively smaller. In contrast, our front-end geometric verification rejects such outliers, allowing the optimization to converge to the true physical solution where the Z-axis error naturally dominates due to the inherent depth-disparity sensitivity of stereo vision. This contrast actually confirms that our geometric verification effectively exposes, rather than conceals, the intrinsic limitations of stereo depth estimation.

To evaluate the sensitivity of the proposed method to the regularization parameter λ in Equation (8), we varied λ from 10^−5^ to 10^2^ and computed the mean and standard deviation of the estimated translations across 30 independent trials. This range encompasses the full spectrum of regularization strengths, from negligible influence of the geometric term (λ = 10^−5^) to its complete dominance in the optimization (λ = 10^2^). As detailed in Table 5, the estimated translation vectors remain virtually identical across this entire range, with variations strictly below 0.001 m. Furthermore, the standard deviations exhibit consistent stability. These results demonstrate that our method is highly robust to the choice of λ. Consequently, we selected λ = 10^−2^ for all subsequent experiments, as it resides comfortably within this stable plateau.

### 3.4. Robot Guidance Performance

To further validate the effectiveness and reliability of the proposed method in real-world automatic charging scenarios, a robot-guided experiment is conducted. Specifically, target alignment control tests for the charging port are executed across 30 distinct initial end-effector poses to assess system performance under varying starting conditions. In each trial, the pose estimated by the proposed method serves as the target for robot motion control, guiding the end-effector to approach the charging port along its surface normal. The ground-truth pose of the charging port was obtained by manual measurement using high-precision calipers and protractors, ensuring that the reference is independent of the vision-based estimation. The end-effector poses were recorded via the teach pendant from real-time joint encoder readings. This same ground-truth reference is used for the pose estimation accuracy evaluation in Section 3.3. The deviation between the final achieved end-effector pose and the ground-truth reference is recorded as the alignment error.

To quantitatively evaluate guidance performance, success criteria are defined as translational errors within ±3 mm for all axes and rotational errors within ±3° for Roll, Pitch, and Yaw. A trial is deemed successful only when all six degrees of freedom simultaneously satisfy these thresholds; otherwise, it is considered a failure.

**Figure 10:**
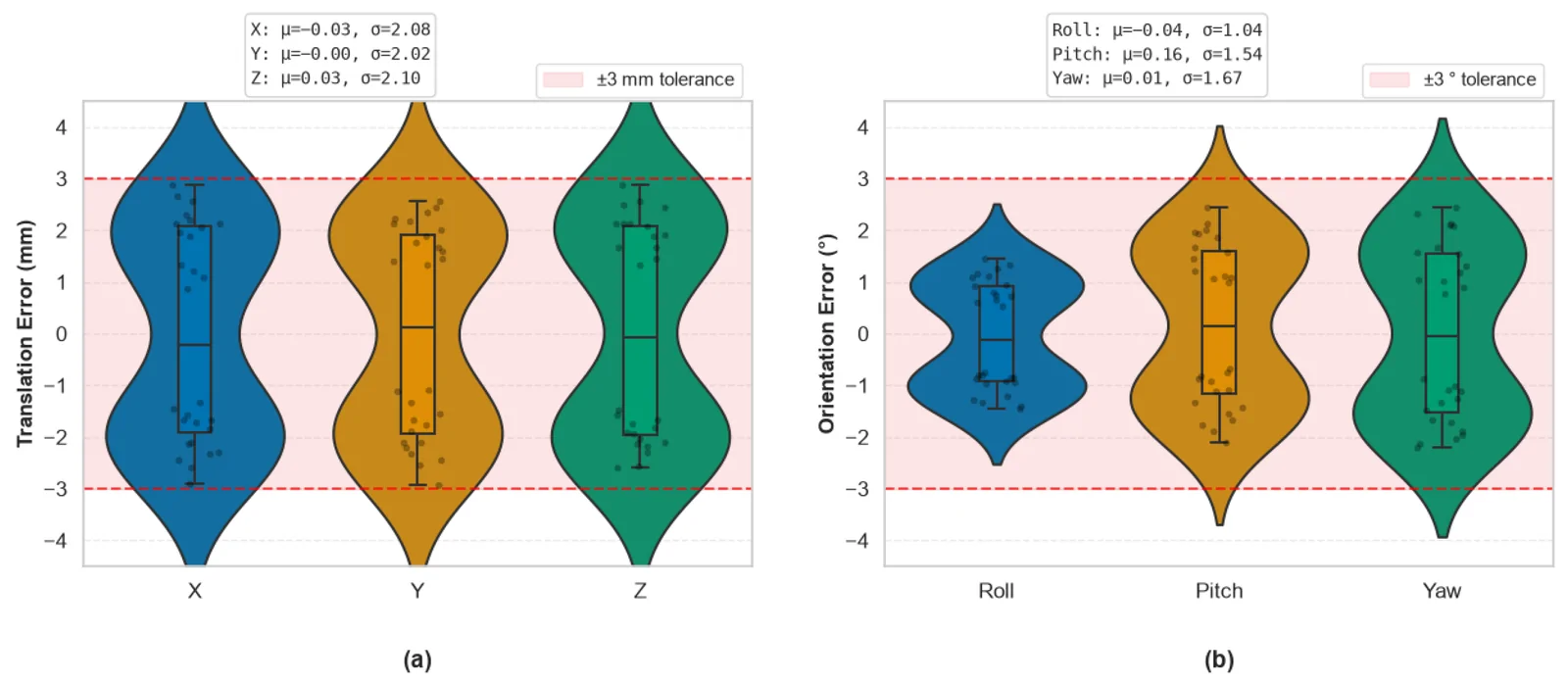
Statistical distribution of alignment errors for the 28 successful robot guidance trials. (**a**) Translational errors along the X, Y, and Z axes; (**b**) Rotational errors for Roll, Pitch, and Yaw angles. The red dotted lines and corresponding colors denote the range that was experimentally verified as successful.

Experimental results demonstrate that 28 of the 30 independent trials achieve precise alignment, achieving a success rate of 93.3%. Figure 10 illustrates the error distributions for these successful cases, where the pink shaded region indicates the allowable tolerance for autonomous charging, boxplots and violin plots depict the statistical and probability density characteristics respectively, and scattered points correspond to individual trial measurements. The two failed trials, detailed in Table 6, exhibit local degree-of-freedom errors exceeding preset thresholds. This failure mode is primarily attributed to specular reflections and occlusions at the charging port edge under specific viewpoints, which degrade feature localization accuracy. Despite these edge cases, the proposed method consistently delivers pose estimates satisfying motion control requirements across the overwhelming majority of operational conditions. It should be noted that the alignment experiment described in this subsection is conducted independently from the pose-estimation accuracy evaluation in Section 3.3. While Section 3.3 characterizes the open-loop estimation error of the proposed algorithm across 30 charging-gun configurations (Figure 8), the present experiment evaluates the final closed-loop alignment accuracy achieved by the robot after motion control, using a separate set of 30 initial end-effector poses (Figure 9). The two error distributions therefore reflect different stages and mechanisms of the overall system and should not be directly compared on a per-axis statistical basis.

Regarding real-time performance, the complete closed-loop cycle remains stable at approximately 78 ms, which encompasses image acquisition, object detection, and robotic motion control. Specifically, the CBAM-YOLOv11 object detection takes about 3 ms, binocular matching and PnP pose estimation require approximately 35 ms, and motion planning and the initial execution delay together consume approximately 40 ms. This response speed fully satisfies the requirements for real-time tracking and adjustment of dynamic targets in autonomous charging scenarios, ensuring a smooth and stable charging process.

Comprehensive experiments confirm that the proposed CBAM-YOLOv11 and geometrically constrained EPnP framework achieves accurate 6-DoF pose estimation and reliably guides robotic alignment with the charging port. These results validate the method’s robustness, repeatability, and engineering applicability, establishing it as a viable visual guidance solution for autonomous EV charging systems.

## 4. Conclusions

This paper presents a comprehensive perception and robotic guidance framework for autonomous EV charging, which addresses the critical challenges of small target detection under severe illumination variations and high-precision 6-DoF pose estimation for texture-less charging ports.

The proposed CBAM-YOLOv11 detector integrates a sequential channel-spatial attention module into the P4 layer of the YOLOv11 backbone, achieving an effective trade-off between computational efficiency and spatial detail preservation. This design significantly enhances feature discriminability for small, texture-less targets while suppressing background clutter and glare. Experimental results demonstrate that our detector achieves 99.2% mAP@0.5, with strict localization metrics mAP@0.75 and mAP@0.5:0.95 improving by 26.2 and 24.6 percentage points over the baseline YOLOv11, confirming the effectiveness of the deliberate attention placement.

For pose estimation, the geometrically constrained EPnP pipeline incorporates front-end topological constraints to purify 2D-3D correspondences, followed by nonlinear refinement with rigid distance priors as regularization. This mechanism reduces the positioning standard deviations along the X, Y, and Z axes to 4.72 mm, 5.65 mm, and 5.60 mm, respectively, approximately 60% lower than conventional EPnP, and also improves rotational stability. The closed-loop system, validated on a physical 6-DoF collaborative robot with a binocular stereo camera, achieves a 93.3% insertion success rate across 30 repeated trials, with a total cycle time of about 78 ms, fully satisfying real-time operational requirements.

Despite these achievements, two failure cases were observed, primarily attributed to specular reflections and occlusions at the charging port edge under extreme viewpoints, which temporarily degraded feature localization. Future work will focus on three directions: (1) integrating temporal information or multi-frame fusion to mitigate single-frame outliers caused by transient glare. (2) exploring end-to-end learning of geometric constraints to further relax manual calibration efforts. (3) expanding the dataset to cover more diverse weather conditions and vehicle models, thereby strengthening generalization for large-scale deployment in real-world toll and parking scenarios. Overall, the proposed framework offers a practical, accurate, and deployable solution for autonomous EV charging systems.

## Figures and Tables

**Table 1:** Training Parameters.

Name	Value
Image Size	640 × 640
Batch	16
Initial Learning Rate	0.01
Weight Decay	0.0005
Box Loss Gain	7.5
Epochs	500

**Table 2:** Quantitative comparison of detection performance among different models. The metrics include Precision, Recall, and mean Average Precision (mAP) at various IoU thresholds.

Model	Precision	Recall	mAP@0.5	mAP@0.75	mAP@0.5:0.95
Baseline	0.968	0.978	0.982	0.598	0.549
Yolov11 + SAM	0.966	0.986	0.985	0.715	0.662
Yolov11 + CAM	0.978	0.983	0.990	0.805	0.748
CBAM-Yolov11 (neck)	0.975	0.988	0.990	0.782	0.735
CBAM-Yolov11 (head)	0.967	0.980	0.987	0.706	0.673
CBAM-Yolov11 (backbone)	0.976	0.988	0.992	0.860	0.795

**Table 3:** 95% confidence intervals for the 6-DoF pose estimation errors of the proposed method.

DOF	σ	Standard Error	95% Confidence Interval	Interval Width	Contains Zero
**Δx (mm)**	4.72	0.862	[−1.69, +1.69]	3.38	Yes
**Δy (mm)**	5.56	1.032	[−2.02, +2.02]	4.04	Yes
**Δz (mm)**	5.60	1.022	[−2.00, +2.00]	4.00	Yes
**Δroll (°)**	0.831	0.152	[−0.298, +0.298]	0.596	Yes
**Δpitch (°)**	2.09	0.382	[−0.748, +0.748]	1.496	Yes
**Δyaw (°)**	1.81	0.330	[−0.647, +0.647]	1.294	Yes

**Table 4:** Quantitative comparison of 6-DoF pose estimation errors (standard deviations) among baseline EPnP, OPnP, and the proposed geometry-constrained method.

Method	X (mm)	Y (mm)	Z (mm)	Roll (°)	Pitch (°)	Yaw (°)
EPnP (Baseline)	16.6	15.5	8.1	1.50	2.10	2.31
OPnP	11.2	10.8	6.9	1.12	2.15	1.98
Proposed method	4.72	5.65	5.60	0.831	2.09	1.81

**Table 5:** Sensitivity analysis of the regularization parameter λ. For each λ value, the mean and standard deviation of the translation vector are computed over 30 independent trials.

λ	Mean tx (m)	Mean ty (m)	Mean tz (m)	Std tx (m)	Std ty (m)	Std tz (m)
10^−5^	0.05221218	−0.00801192	0.18615548	0.03450335	0.03066073	0.02991938
10^−4^	0.05221177	−0.00801187	0.18615698	0.03450311	0.03066060	0.02991947
10^−3^	0.05221252	−0.00801143	0.18615445	0.03450435	0.03066084	0.02991938
10^−2^	0.05221255	−0.00801158	0.18615483	0.03450420	0.03066062	0.02991930
10^−1^	0.05221256	−0.00801158	0.18615485	0.03450420	0.03066062	0.02991930
10^0^	0.05221256	−0.00801157	0.18615485	0.03450420	0.03066062	0.02991930
10^1^	0.05221256	−0.00801156	0.18615485	0.03450420	0.03066062	0.02991930
10^2^	0.05221256	−0.00801157	0.18615483	0.03450420	0.03066062	0.02991930

**Table 6:** Exceedance of alignment errors beyond the preset thresholds in failed trials.

Group	X (mm)	Y (mm)	Z (mm)	Roll (°)	Pitch (°)	Yaw (°)
1	1.88	−2.11	4.62	1.12	−2.21	2.44
2	−2.44	2.88	−2.31	3.42	−3.15	2.12

## Data Availability

The data are unavailable due to privacy restrictions. The original data presented in the study are openly available in Roboflow Universe at https://app.roboflow.com/ccs1-djso8/charging-port-013de/1 (accessed on 1 March 2026).
